# Cereal-based solid-state production and storage stability of native *Trichoderma harzianum* isolates for biological control applications

**DOI:** 10.3389/fmicb.2026.1991071

**Published:** 2026-10-14

**Authors:** Dieumerci Dramani Londjiringa, Faith Mabiki, Richard Madege

**Affiliations:** 1 Department of Crop Science and Horticulture, Sokoine University of Agriculture, Morogoro, Tanzania; 2 Department of Chemistry and Physics, Sokoine University of Agriculture, Morogoro, Tanzania

**Keywords:** *Trichoderma harzianum*, spore production, storage stability, cereal substrates, bioformulation

## Abstract

The effectiveness of *Trichoderma*-based biocontrol products depends on achieving high spore yields and maintaining viable propagules during storage. This study evaluated the effects of substrate type, isolate, and storage duration on spore production and viability of four *Trichoderma harzianum* isolates (BH10-2, KH5-1, MG7-2, and SH11-2) cultured on four cereal-based substrates: rice grain (RG), rice powder (RP), sorghum grain (SG), and sorghum powder (SP). The experiment was arranged in a factorial Completely Randomized Design (CRD) with three replications under laboratory conditions. Spore production was assessed 10 days after fermentation, while spore viability was determined after 30, 60, and 90 days of storage following a 7-day drying period. Colony-forming unit (CFU) data were log-transformed before analysis using two-way ANOVA for initial spore production and a linear mixed-effects model (LMM) for repeated measurements of spore viability during storage. Mean separation was performed using Tukey's HSD test at *P* ≤ 0.05. Initial spore production was significantly influenced by the interaction between isolate and substrate (*P* < 0.05). Rice powder supported the highest spore production, with isolate BH10-2 producing the greatest spore yield (8.55 log CFU g^−1^), whereas isolate MG7-2 grown on sorghum grain recorded the lowest initial spore production (5.81 log CFU g^−1^). Spore viability was significantly affected by isolate, substrate, storage duration, and their interactions (*P* < 0.05). Spore viability increased from Day 10 to Day 30, indicating continued sporulation during the early storage phase, before declining progressively through Days 60 and 90. Sorghum grain consistently maintained the highest spore viability during prolonged storage, particularly for isolate MG7-2, whereas rice powder exhibited the greatest reduction in viability after 90 days. Overall, the results demonstrate that substrate selection influences both initial sporulation and long-term storage stability of *Trichoderma*. The combination of isolate MG7-2 and sorghum grain showed the greatest potential for developing stable *Trichoderma*-based bioformulations suitable for sustainable biological control.

## 1 Introduction

The rising demand for sustainable farming methods has heightened interest in eco-friendly alternatives to synthetic chemicals. Among these, *Trichoderma* species are among the most extensively studied microbial agents due to their dual roles as biological control agents and promoters of plant growth (Yao et al., 2023; Guzmán-Guzmán et al., 2023; Asghar et al., 2024). These fungi can inhibit many soil-borne pathogens through mechanisms such as mycoparasitism (Theradimani et al., 2024; Mukherjee et al., 2022), antibiosis (Manzar et al., 2022), and competition for nutrients and space (Oyesola et al., 2025; Theradimani et al., 2024). Moreover, *Trichoderma* strains enhance plant development by improving nutrient uptake, promoting root growth, and triggering systemic resistance, making them important tools in sustainable crop production.

The increasing use of *Trichoderma*-based products in agriculture has created a need for effective and economical methods for their large-scale production (Mattedi et al., 2023). One of the main challenges in commercializing microbial biocontrol agents is developing formulations that ensure high spore yields, viability, and stability during storage and application. Liquid fermentation systems, although effective, often require sophisticated infrastructure and incur high operational costs, which may be infeasible in many developing regions (Lyubenova et al., 2023). Consequently, solid-state fermentation (SSF) has gained considerable attention as a practical and economically viable alternative for the mass production of fungal biocontrol agents (Kurpnieks et al., 2023; Mattedi et al., 2023).

Solid substrates such as cereal grains and agro-industrial by-products serve as both nutritional sources and physical platforms for fungal growth and sporulation (Rai et al., 2023; Naeimi et al., 2020). Materials such as rice and sorghum are particularly attractive because they are inexpensive, readily available, and capable of supporting fungal colonization. However, their physicochemical characteristics, including particle size, moisture-holding capacity, nutrient composition, porosity, and aeration, strongly influence fungal growth, conidiation, and formulation quality (Mulatu et al., 2021; Alias et al., 2022; Sala et al., 2021; Cavalcante et al., 2008). Grain substrates generally provide better aeration and structural integrity than powdered substrates, whereas powdered materials offer greater surface area and nutrient accessibility that may favor rapid sporulation but can reduce aeration and long-term stability (Alias et al., 2022; Bulgari et al., 2023). Understanding these differences is essential for selecting substrates that maximize both spore production and storage stability.

Maintaining high spore viability during storage is equally important because the effectiveness of *Trichoderma*-based bioformulations depends on the survival of viable propagules until application. Previous studies have shown that storage stability is influenced by substrate characteristics, moisture content, drying methods, and storage conditions (Pedreschi and Aguilera, 1997; Fernández-Sandoval et al., 2012). Furthermore, cultivation media and formulation components can influence colony-forming units during storage by affecting fungal biomass production and propagule survival (Rimkus et al., 2023). Substrate selection also plays a crucial role in shelf life, with studies demonstrating significant differences in spore survival among organic and cereal-based substrates during storage (Kamboj et al., 2023; Surve, 2024). These findings highlight the importance of identifying substrates that not only promote high initial sporulation but also preserve spore viability during prolonged storage.

Despite these advances, information on the performance of native Tanzanian *Trichoderma harzianum* isolates grown on locally available cereal substrates remains limited. Most previous studies have focused either on commercial strains or on a single substrate type, with little attention given to comparing grain and powdered cereal substrates for both spore production and long-term storage stability (Naeimi et al., 2020; Mulatu et al., 2021; Kamboj et al., 2023). Such comparisons are particularly important because particle size may differentially influence fungal colonization, aeration, nutrient availability, and spore persistence (Alias et al., 2022; Bulgari et al., 2023; Sala et al., 2021). Moreover, developing efficient production systems based on inexpensive and locally available agricultural materials is essential for reducing production costs and increasing the accessibility of *Trichoderma*-based biocontrol products for farmers in Tanzania and other resource-limited regions. We hypothesized that substrate composition and physical form (grain vs. powder) influence both sporulation and long-term viability of native *Trichoderma harzianum* isolates. Therefore, this study evaluated the effects of four cereal-based substrates: rice grain, rice powder, sorghum grain, and sorghum powder on spore production and storage viability of four native *T. harzianum* isolates, with the aim of identifying the most suitable isolate–substrate combination for the development of stable and cost-effective bioformulations.

## 2 Materials and methods

### 2.1 Study area and source of isolates

The study was conducted under laboratory conditions in the Plant Pathology laboratory at Sokoine University of Agriculture, Tanzania. Four isolates of *Trichoderma harzianum*, previously obtained from different agro-ecological zones of Tanzania and maintained on slants at 4 °C, were used in this study: MG7-2, BH10-2, KH5-1, and SH11-2 (Nyakeko et al., 2026a,b). The isolates were sub-cultured on potato dextrose agar (PDA) and incubated at 25 ± 2 °Cfor 5–7 days prior to use.

### 2.2 Cereal-based substrates

This study evaluated four cereal-based substrates: rice grain (RG), rice powder (RP), sorghum grain (SG), and sorghum powder (SP). Rice and sorghum grains were purchased in their dry form. To prepare the powdered substrates, the grains were separately soaked in water at a 1:2 (w/v) ratio for 30 min to soften them. The excess water was removed using a strainer, and the softened grains were then ground using a mixer grinder to obtain a coarse powder. The resulting rice powder (RP) and sorghum powder (SP) were subsequently used as substrates for Trichoderma cultivation. This procedure was adapted from the method reported by Rini et al. (2018).

### 2.3 Solid-state fermentation

Before inoculation, all substrates were soaked in tap water overnight, and excess water was drained (Dhruw et al., 2023). This soaking step was performed to adjust the moisture content of the substrates prior to fermentation. Then, 50 g of each substrate was placed in autoclavable containers and sterilized by autoclaving at 121 °C for 20 min on three successive days (Naeimi et al., 2020). The substrate was sterilized via a three-cycle autoclaving process on consecutive days to eliminate heat-resistant spore-forming bacteria that survive a single sterilization cycle, thereby preventing competitive microbial contamination during fungal colonization. After cooling the substrates to room temperature, three 5 mm mycelial discs cut from the edge of 7-day-old PDA cultures of each *Trichoderma* isolate were inoculated into each substrate under aseptic conditions to ensure uniform colonization, and the substrates were then kept at ambient laboratory temperature for 10 days, with periodic hand shaking to promote uniform growth. After incubation, the colonized substrates were removed from the containers, air-dried for up to 7 days in a Nalgene Laboratory Desiccator to reduce moisture content before packaging in sealed polyethene bags for storage. The bags were stored in a covered cardboard box under ambient laboratory conditions for 90 days to protect them from direct light and minimize environmental fluctuations. The bags remained unopened throughout the storage period except during sampling for spore viability assessment. Room temperature and relative humidity were monitored daily using a digital thermo-hygrometer.

### 2.4 Spore production after 10 days of fermentation

We determined the number of viable *Trichoderma* propagules using a serial dilution and spread-plate method, following the approach described by Naeimi et al. (2020) and Rini et al. (2018). Briefly, 1 g of each colonized substrate was aseptically suspended in 9 mL of sterile distilled water and vigorously vortexed for 2–3 min. We then prepared serial ten-fold dilutions and spread 0.1 mL aliquots from appropriate dilutions onto PDA plates. We incubated the plates at room temperature for 2 days, then counted colonies. The number of viable propagules was expressed as colony-forming units per gram of substrate (CFU g^−1^), calculated as follows:


$$\begin{array}{l} CFU/g\;=\frac{N\times \;D\;}{V\times \;W} \end{array}$$

where N = number of colonies counted, D = dilution factor, V = volume plated (mL), and W = sample weight (g).

### 2.5 Assessment of spore viability during storage

Spore viability was assessed after 30, 60, and 90 days of storage using the serial dilution and spread-plate method described in Section 2.4. At each sampling time, viable Trichoderma propagules were quantified and expressed as CFU g^−1^. Daily temperature and relative humidity were monitored and recorded throughout the storage period to characterize the storage conditions.

### 2.6 Experimental design

The experiment was set up using a factorial Completely Randomized Design (CRD) with four substrates and four *Trichoderma* isolates, each treatment being replicated three times. Spore counts were taken at every sampling point.

### 2.7 Data analysis

Data analysis was performed using R (version 4.5.0). Spore production data collected 10 days after fermentation were analyzed using a two-way analysis of variance (ANOVA) to evaluate the effects of substrate type, isolate, and their interaction. Spore viability data collected after 30, 60, and 90 days of storage were analyzed using a linear mixed-effects model (LMM), with isolate, substrate, storage duration, and their interactions treated as fixed effects, and replicate as a random effect to account for repeated measurements over time. Colony-forming unit (CFU) data were log-transformed into log10 CFU g^−1^ before analysis to improve normality and homogeneity of variances. Pairwise comparisons of treatment means were performed using Tukey's Honest Significant Difference (HSD) test at the 5% significance level. The assumptions of normality and homogeneity of variance for the ANOVA were assessed using the Shapiro–Wilk and Levene's tests, respectively. Estimated marginal means for the mixed-effects model were obtained using the emmeans package, and compact letter displays were used to separate treatment means. Descriptive statistics, including means and standard deviations, were calculated for all treatment combinations.

## 3 Results

### 3.1 Storage environmental conditions

Room temperature and relative humidity varied during the first 30 days of storage. During this period, room temperature fluctuated between approximately 25 and 32 °C, while relative humidity ranged from about 43% to 63% (Figure 1). After 30 days, both environmental parameters became relatively stable, with temperature maintained between 26.5 and 27.0 °Cand relative humidity between 72% and 74% for the remainder of the storage period.

**Figure 1:**
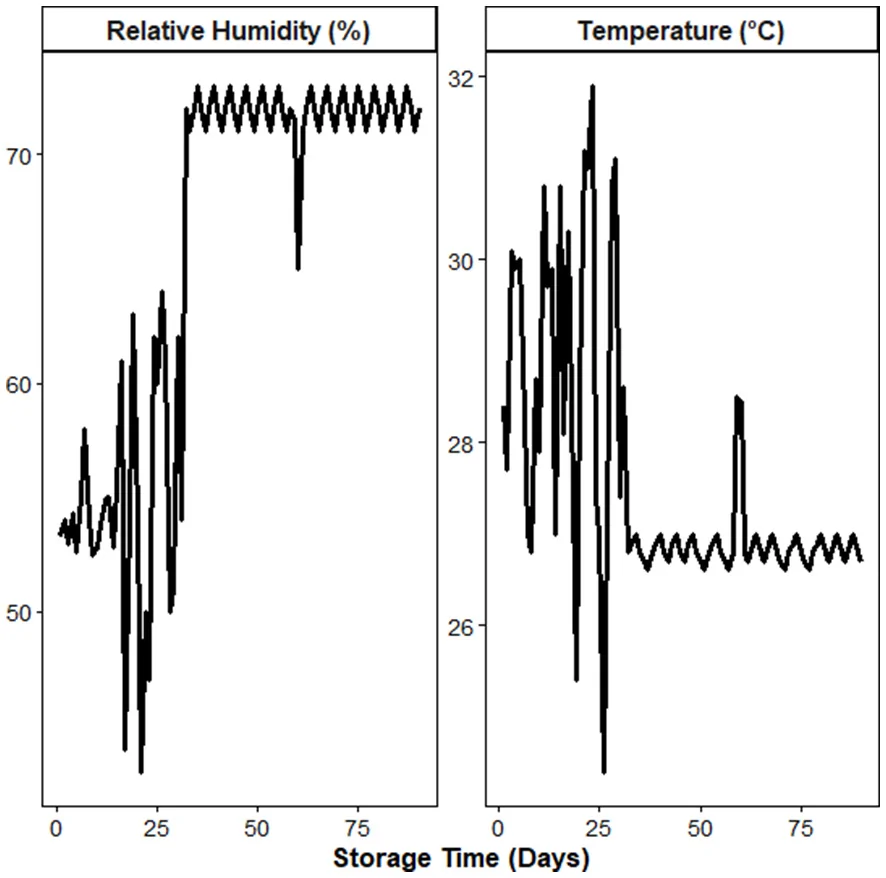
Variation in temperature (°C) and relative humidity (%) during the 90-day storage period.

### 3.2 Initial spore production after 10 days

#### 3.2.1 Effect of isolate on spore production

Spore production differed significantly among the four *Trichoderma* isolates (*P* < 0.05). Isolate BH10-2 produced the highest mean spore count (7.31 log CFU g^−1^) and was significantly superior to MG7-2 (6.75 log CFU g^−1^), but not significantly different from KH5-1 (7.07 log CFU g^−1^) and SH11-2 (6.91 log CFU g^−1^). MG7-2 recorded the lowest spore production (Figure 2).

**Figure 2:**
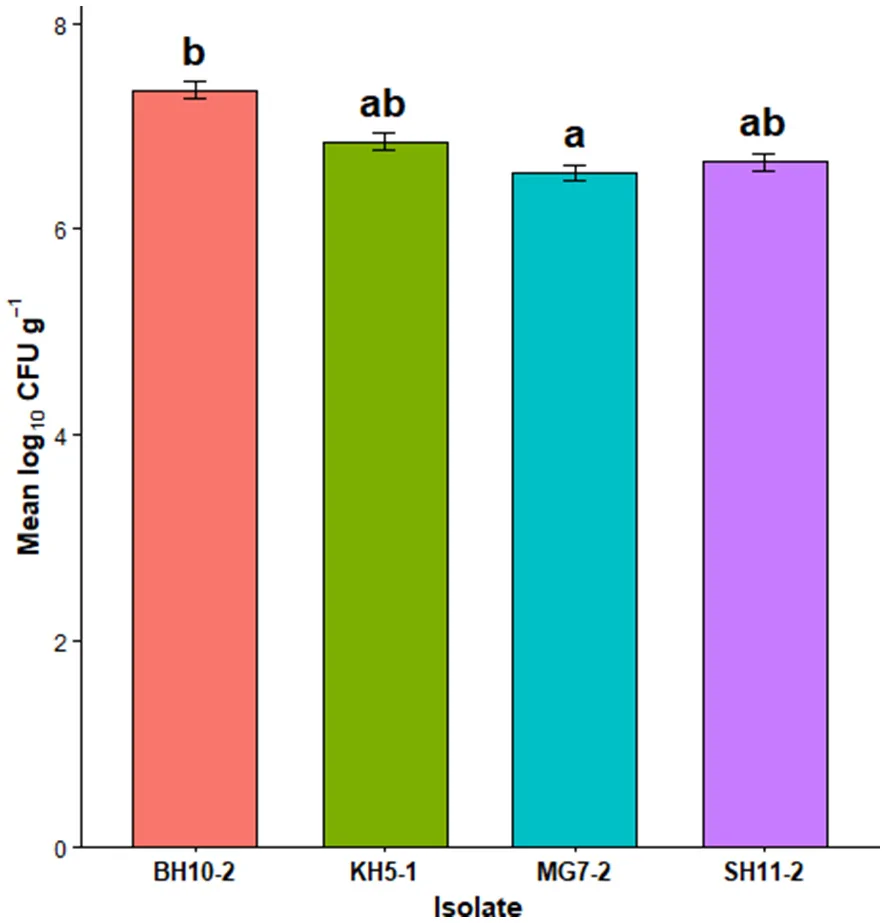
Effect of isolate on spore production of *Trichoderma* isolates expressed as mean log CFU g^−1^ ± SD. Bars with different letters indicate significant differences according to Tukey's HSD test at *P* ≤ 0.05.

#### 3.2.2 Effect of substrate on spore production

Substrate type significantly influenced spore production after 10 days (*P* < 0.05). Rice powder (RP) supported the highest mean spore production (7.90 log CFU g^−1^) and was significantly superior to all other substrates. Sorghum grain (SG) produced the lowest spore count (6.19 log CFU g^−1^), whereas rice grain (RG) (6.96 log CFU g^−1^) and sorghum powder (SP) (6.98 log CFU g^−1^) showed intermediate spore production and did not differ significantly from each other (Figure 3).

**Figure 3:**
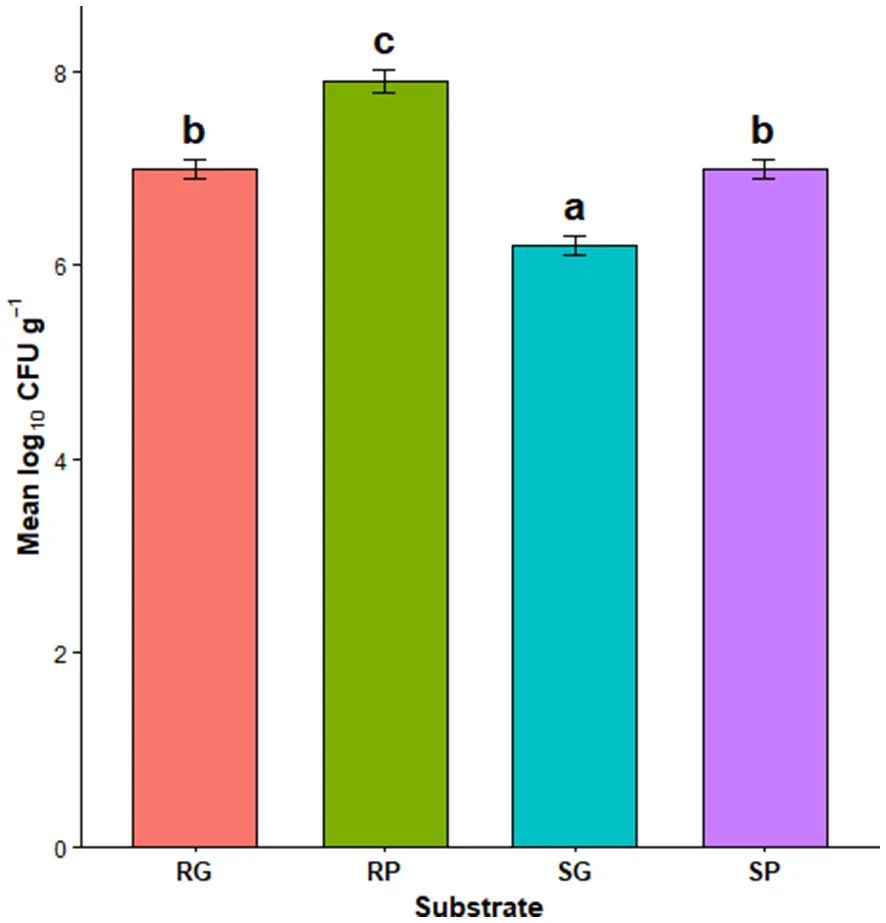
Effect of substrate on spore production, expressed as mean log CFU g^−1^ ± SD. Bars with different letters indicate significant differences according to Tukey's HSD test at *P* ≤ 0.05. RG, rice grains, SG, sorghum grains, SP, sorghum powder and RP, rice powder.

#### 3.2.3 Interaction effect of isolate × substrate on spore production

The interaction between isolate and substrate significantly affected spore production after 10 days (P < 0.05). The highest spore production was recorded for isolate BH10-2 grown on rice powder (RP) (8.55 ± 0.05 log CFU g^−1^), followed by KH5-1 on RP (8.27 ± 1.14 log CFU g^−1^). In contrast, the lowest spore production was observed for isolate MG7-2 grown on sorghum grain (SG) (5.81 ± 0.02 log CFU g^−1^) (Table 1).

**Table 1:** Interaction effect of isolate × substrate on spore production expressed as mean log CFU g^−1^ ± standard deviation (SD).

Isolate × Substrate	Mean ±SD
MG7-2 × SG	5.81 ± 0.02^a^
KH5-1 × RG	5.82 ± 0.51^a^
SH11-2 × SG	5.90 ± 0.04^a^
BH10-2 × SG	6.25 ± 0.10^ab^
MG7-2 × SP	6.66 ± 0.03^abc^
SH11-2 × SP	6.74 ± 0.03^abc^
KH5-1 × SG	6.76 ± 0.08^abc^
BH10-2 × SP	7.09 ± 0.58^bc^
MG7-2 × RG	7.22 ± 0.12^bcd^
MG7-2 × RP	7.31 ± 0.11^bcd^
BH10-2 × RG	7.34 ± 0.02^bcd^
KH5-1 × SP	7.42 ± 0.05^cde^
SH11-2 × RP	7.47 ± 0.56^cde^
SH11-2 × RG	7.48 ± 0.05^cde^
KH5-1 × RP	8.27 ± 1.14^de^
BH10-2 × RP	8.55 ± 0.05^e^
P value	1.04 × 10^−5^

Means followed by different letters are significantly different according to Tukey's HSD test at *p* ≤ 0.05.

RG, rice grains, SG, sorghum grains, SP, sorghum powder and RP, rice powder.

### 3.3 Spore viability during storage

#### 3.3.1 Effect of isolate on spore viability

Isolate significantly affected *Trichoderma* spore viability during storage (P < 0.05). Isolate MG7-2 exhibited the highest mean spore viability (8.69 log CFU g^−1^) and was significantly superior to all other isolates. BH10-2 (8.48 log CFU g^−1^) and KH5-1 (8.45 log CFU g^−1^) showed intermediate spore viability and did not differ significantly from each other. SH11-2 recorded the lowest mean spore viability (8.04 log CFU g^−1^), which was significantly lower than that of the other isolates (Figure 4).

**Figure 4:**
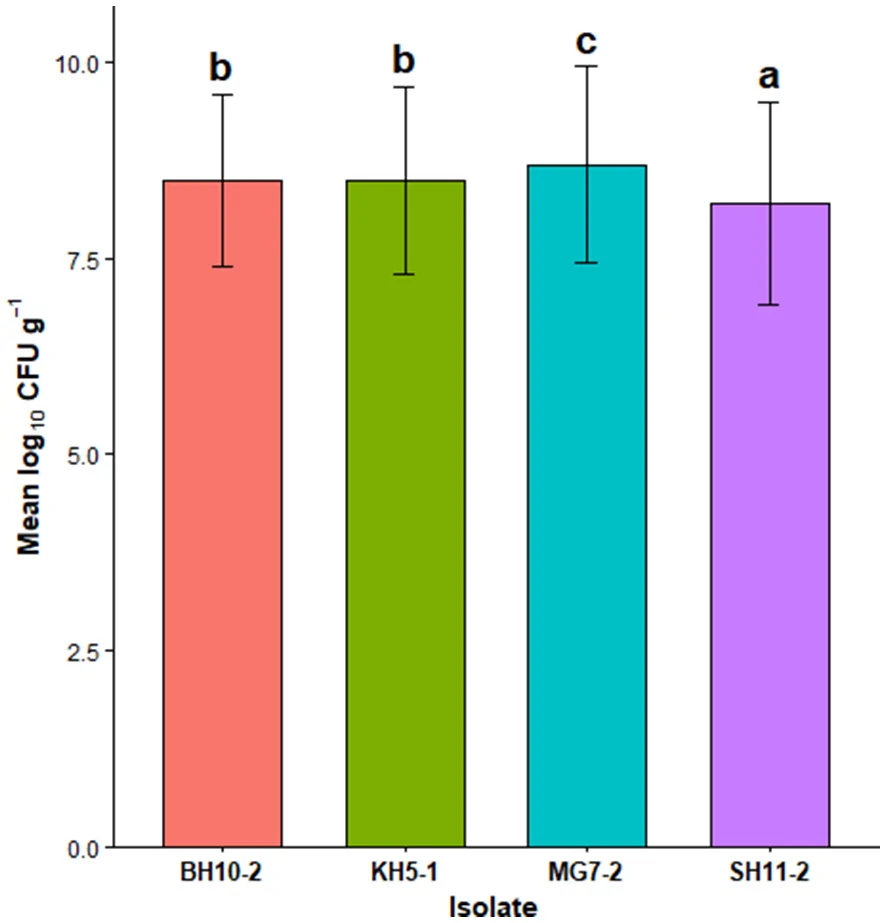
Effect of isolate on spore viability during storage expressed as mean log CFU g^−1^ ± SD. Bars with different letters indicate significant differences according to Tukey's HSD test at *P* ≤ 0.05.

#### 3.3.2 Effect of substrate on spore viability

Substrate significantly affected spore viability during storage (P < 0.05). Sorghum grain (SG) supported the highest mean spore viability (9.02 log CFU g^−1^) and was significantly superior to all other substrates. Rice grain (RG) exhibited intermediate spore viability (8.60 log CFU g^−1^), which was significantly higher than that of rice powder (RP) (8.13 log CFU g^−1^) and sorghum powder (SP) (8.03 log CFU g^−1^) (Figure 5).

**Figure 5:**
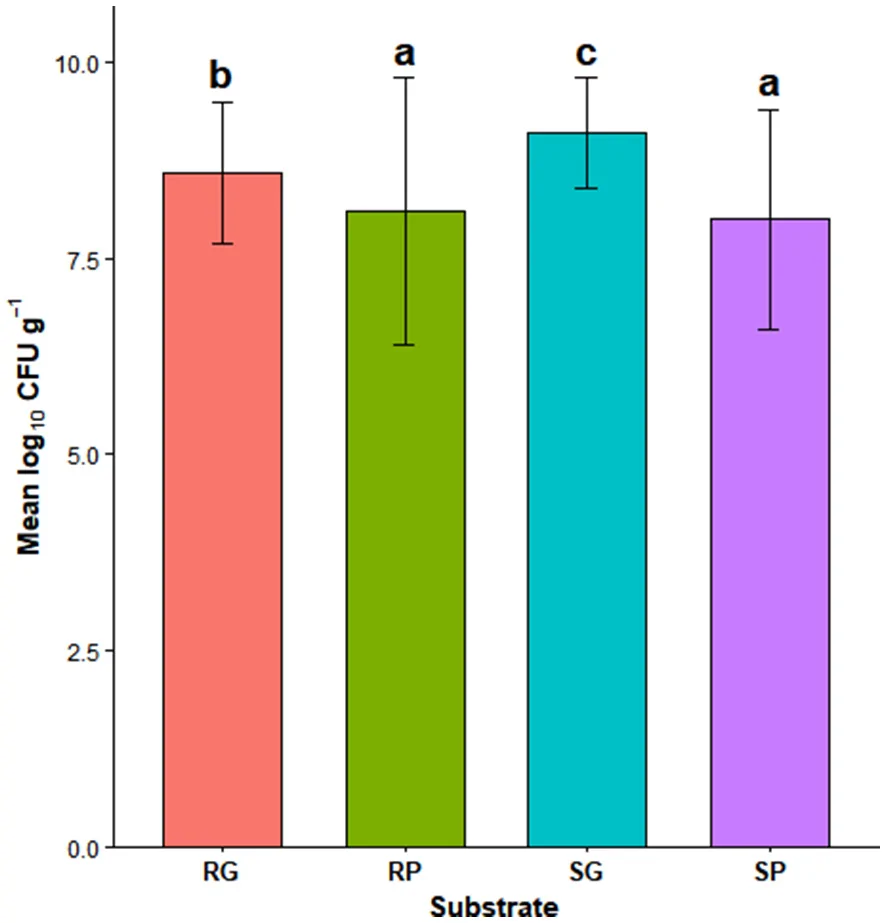
Effect of substrate on spore viability during storage, expressed as mean log CFU g^−1^ ± SD. Bars with different letters indicate significant differences according to Tukey's HSD test at *P* ≤ 0.05. RG, rice grains, SG, sorghum grains, SP, sorghum powder and RP, rice powder.

#### 3.3.3 Effect of storage duration on spore viability

Storage duration significantly affected *Trichoderma* spore viability (P < 0.05). The highest mean spore viability was recorded after 30 days of storage (9.68 log CFU g^−1^), followed by 60 days (8.61 log CFU g^−1^), with the lowest viability observed after 90 days of storage (7.11 log CFU g^−1^) (Figure 6).

**Figure 6:**
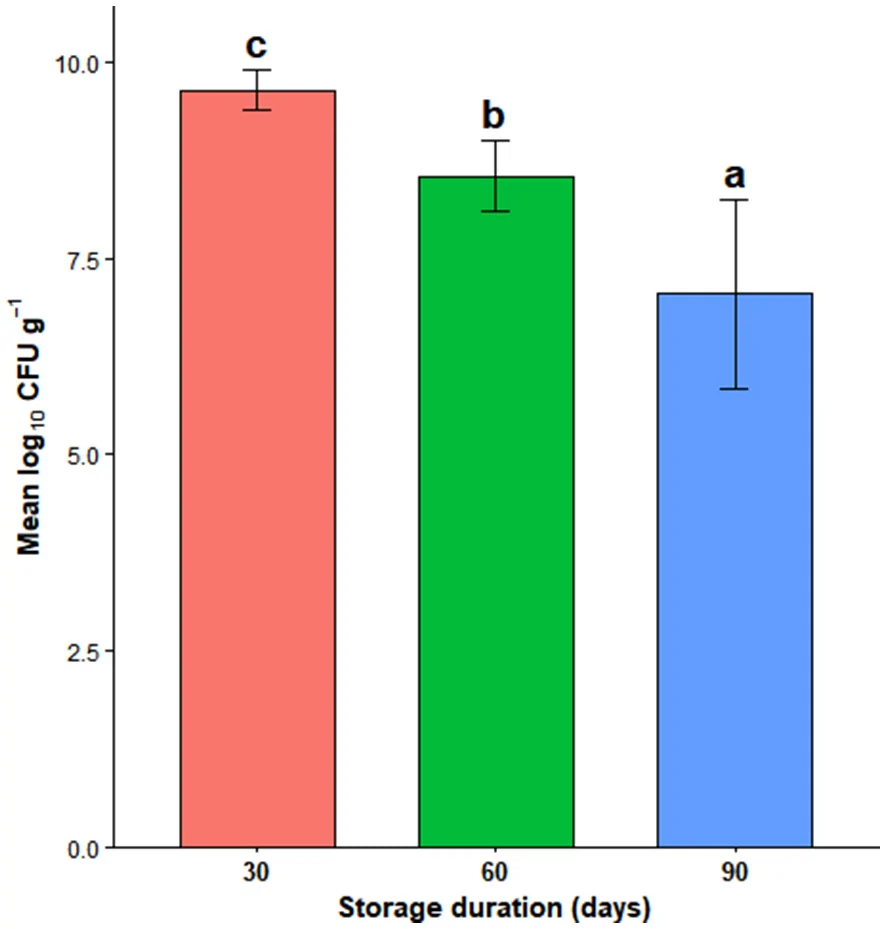
Effect of storage duration on spore viability expressed as mean log CFU g^−1^ ± SD. Bars with different letters indicate significant differences according to Tukey's HSD test at *P* ≤ 0.05.

#### 3.3.4 Interaction effect of isolate × substrate on spore viability

The interaction between isolate and substrate significantly affected spore viability during storage (P < 0.05), indicating that substrate performance varied with the *Trichoderma* isolate. The highest spore viability was recorded for isolate MG7-2 grown on sorghum grain (SG) (9.56 ± 0.17 log CFU g^−1^), followed by BH10-2 on SG (9.28 ± 0.39 log CFU g^−1^). In contrast, the lowest spore viability was observed for BH10-2 grown on sorghum powder (SP) (7.88 ± 1.54 log CFU g^−1^) and rice powder (RP) (7.95 ± 1.93 log CFU g^−1^), which did not differ significantly (Table 2).

**Table 2:** Interaction effect of *Trichoderma* isolates and solid substrates on spore viability.

Isolate × Substrate	Mean ±SD
BH10-2 × RG	8.79 ± 0.94^d^
BH10-2 × RP	7.95 ± 1.93^a^
BH10-2 × SG	9.28 ± 0.39^ef^
BH10-2 × SP	7.88 ± 1.54^a^
KH5-1 × RG	8.56 ± 0.99^cd^
KH5-1 × RP	8.17 ± 1.60^abc^
KH5-1 × SG	8.92 ± 0.57^de^
KH5-1 × SP	8.14 ± 1.17^abc^
MG7-2 × RG	8.78 ± 0.99^d^
MG7-2 × RP	8.26 ± 1.69^abc^
MG7-2 × SG	9.56 ± 0.17^f^
MG7-2 × SP	8.11 ± 1.49^abc^
SH11-2 × RG	8.23 ± 0.90^abc^
SH11-2 × RP	8.14 ± 1.72^abc^
SH11-2 × SG	8.44 ± 0.78^bcd^
SH11-2 × SP	8.01 ± 1.72^ab^
P value	1.518 × 10^−5^

Values are presented as mean ± standard deviation. Means followed by different letters are significantly different according to Tukey's HSD test at *p* ≤ 0.05.

RG: rice grains, SG: sorghum grains, SP: sorghum powder and RP: rice powder.

#### 3.3.5 Interaction effect of isolate × storage duration on spore viability

The interaction between isolate and storage duration significantly influenced spore viability (*P* < 0.05). All isolates exhibited the highest spore viability after 30 days of storage (9.57–9.76 log CFU g^−1^), with no significant differences among them. Spore viability declined significantly after 60 days and decreased further after 90 days. At 90 days, MG7-2 maintained the highest spore viability (7.35 ± 1.42 log CFU g^−1^), whereas SH11-2 recorded the lowest viability (6.71 ± 0.87 log CFU g^−1^) (Table 3).

**Table 3:** Interaction effect of *Trichoderma* isolates and storage duration on spore viability.

Isolate × Storage duration	Mean ±SD
BH10-2 × 30 days	9.76 ± 0.09^e^
BH10-2 × 60 days	8.60 ± 0.56^cd^
BH10-2 × 90 days	7.07 ± 1.42^ab^
KH5-1 × 30 days	9.58 ± 0.27^e^
KH5-1 × 60 days	8.55 ± 0.39^c^
KH5-1 × 90 days	7.22 ± 0.93^b^
MG7-2 × 30 days	9.72 ± 0.21^e^
MG7-2 × 60 days	8.95 ± 0.48^d^
MG7-2 × 90 days	7.35 ± 1.42^b^
SH11-2 × 30 days	9.57 ± 0.30^e^
SH11-2 × 60 days	8.34 ± 0.29^c^
SH11-2 × 90 days	6.71 ± 0.87^a^
*P* value	0.004

Values are presented as mean ± standard deviation.

Means followed by different letters are significantly different according to Tukey's HSD test at *p* ≤ 0.05.

#### 3.3.6 Interaction effect of substrate × storage duration on spore viability

The interaction between substrate and storage duration significantly affected spore viability (P < 0.05). All substrates maintained similarly high spore viability after 30 days (9.53–9.88 log CFU g^−1^), with no significant differences among them. Spore viability declined after 60 days and decreased further after 90 days. At 90 days, sorghum grain (SG) maintained the highest spore viability (8.63 ± 0.70 log CFU g^−1^), followed by rice grain (RG) (7.49 ± 0.27 log CFU g^−1^), whereas rice powder (RP) (5.99 ± 0.48 log CFU g^−1^) and sorghum powder (SP) (6.25 ± 0.47 log CFU g^−1^) exhibited the lowest viability (Table 4).

**Table 4:** Interaction effect of substrate type and storage duration on spore viability.

Substrate × Storage duration	Mean ±SD
RG × 30 days	9.68 ± 0.29^e^
RG × 60 days	8.61 ± 0.21^cd^
RG × 90 days	7.49 ± 0.27^b^
RP × 30 days	9.88 ± 0.07^e^
RP × 60 days	8.52 ± 0.31^c^
RP × 90 days	5.99 ± 0.48^a^
SG × 30 days	9.54 ± 0.16^e^
SG × 60 days	8.98 ± 0.63^d^
SG × 90 days	8.63 ± 0.70^cd^
SP × 30 days	9.53 ± 0.22^e^
SP × 60 days	8.34 ± 0.46^c^
SP × 90 days	6.25 ± 0.47^a^
*P* value	2.2 × 10^−16^

Values are presented as mean ± standard deviation. Means followed by different letters are significantly different according to Tukey's HSD test at *p* ≤ 0.05.

RG, rice grains, SG, sorghum grains, SP, sorghum powder and RP: rice powder.

#### 3.3.7 Three-way interaction effect of isolate × substrate × storage duration on spore viability

The three-way interaction between isolate, substrate, and storage duration significantly affected spore viability (*P* < 0.05). Regardless of the isolate or substrate, spore viability generally declined with increasing storage duration. Among the substrates, sorghum grain (SG) consistently maintained the highest spore viability throughout the 90-day storage period, particularly for isolate MG7-2. In contrast, rice powder (RP) and sorghum powder (SP) exhibited the greatest decline in spore viability after 90 days, whereas rice grain (RG) showed an intermediate level of viability (Figures 7, 8).

**Figure 7:**
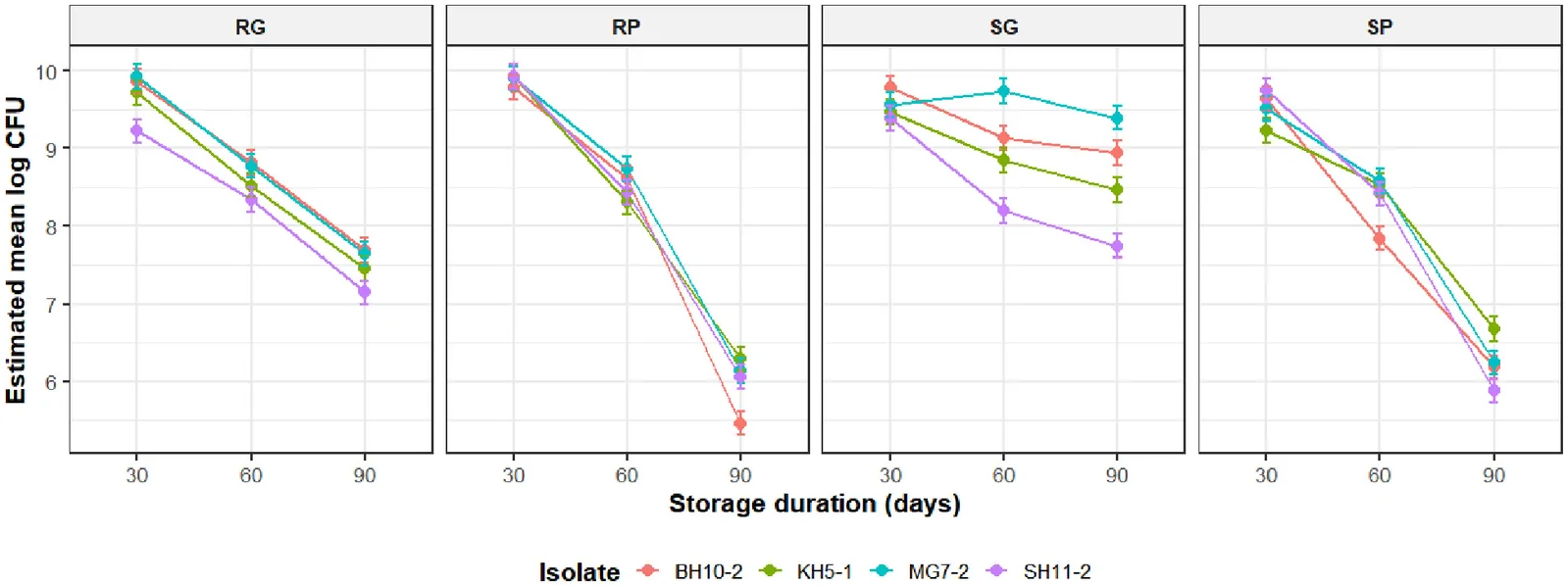
Three-way interaction effect of *Trichoderma* isolate, substrate, and storage duration on spore viability. Means ± SD of spore viability (log CFU g^−1^) for four *Trichoderma* isolates (BH10-2, KH5-1, MG7-2, and SH11-2) grown on four substrates, Rice grain (RG), Rice powder (RP), Sorghum grain (SG), and Sorghum powder (SP) and stored for 30, 60, and 90 days. Points connected by lines show changes in spore viability over the storage period for each isolate.

**Figure 8:**
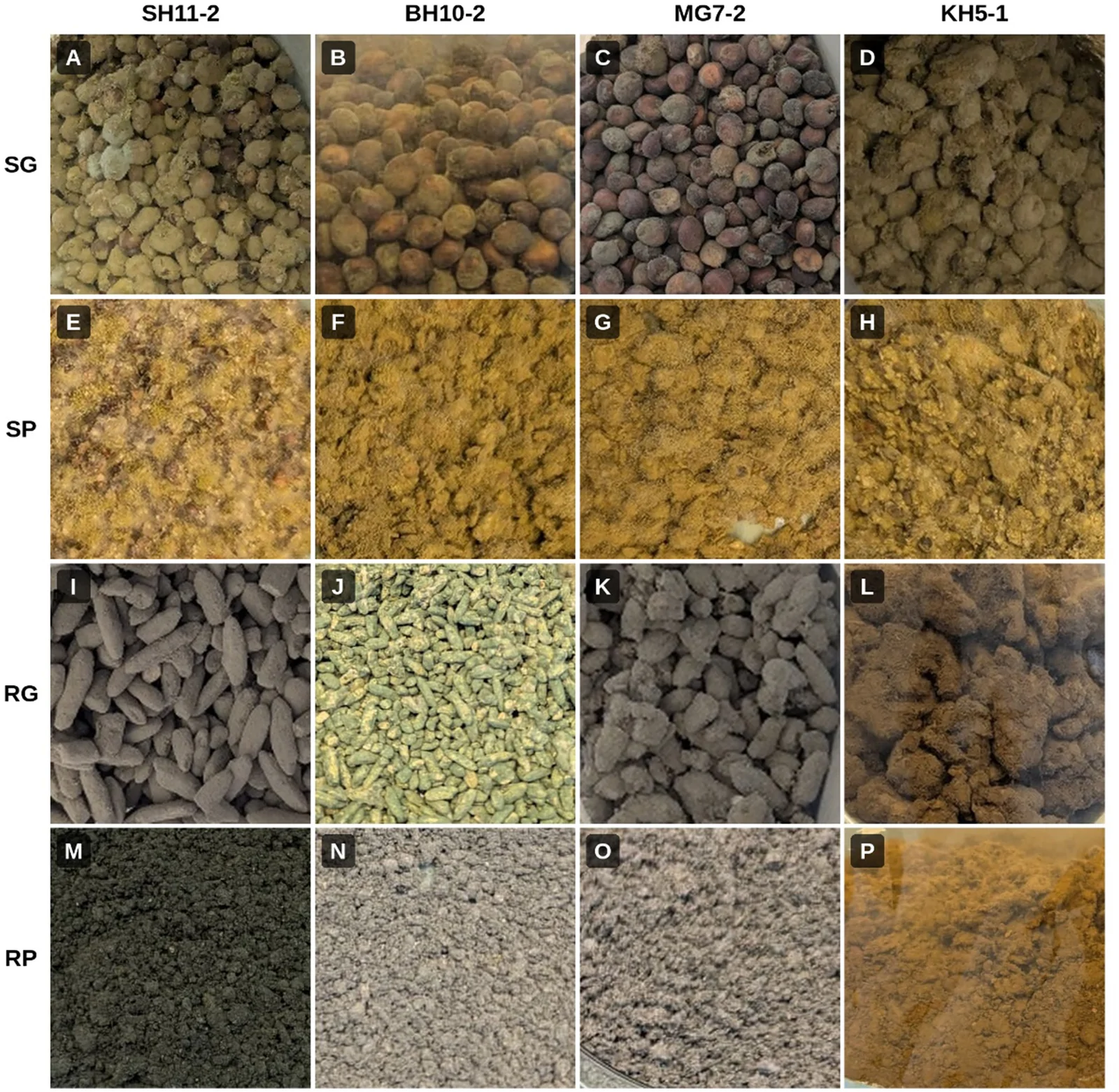
Morphological appearance of *Trichoderma harzianum* isolates BH10-2, KH5-1, MG7-2, and SH11-2 on four cereal-based substrates after 90 days of storage. Columns represent isolates and rows represent substrates: Sorghum grain (SG), and Sorghum powder (SP), Rice grain (RG), Rice powder (RP). Panels (**A–P**) correspond to the respective isolate–substrate combinations.

## 4 Discussion

This study demonstrated that spore production and long-term viability of *Trichoderma* isolates were significantly influenced by isolate type, substrate composition, and storage duration, with significant interaction effects among these factors. These findings indicate that successful development of *Trichoderma*-based bioformulations depends not only on maximizing initial spore production but also on selecting suitable isolate–substrate combinations capable of maintaining propagule viability during storage. Similar findings have been reported previously, showing that fungal sporulation, formulation stability, and shelf life are strongly affected by substrate characteristics and isolate-specific physiological traits (Boblina et al., 2020; Rajput and Shahzad, 2015; Kamboj et al., 2023).

Significant differences among isolates were observed for both initial spore production and subsequent spore viability, reflecting inherent genetic and physiological variation among *Trichoderma* strains. Although BH10-2 produced the highest initial spore concentration on rice powder (RP), isolate MG7-2 consistently maintained the greatest spore viability throughout the storage period, suggesting superior tolerance to storage-associated stresses. Such differences have been widely reported among *Trichoderma* isolates and are often associated with variation in growth characteristics, environmental stress tolerance, and biocontrol performance (Nandini et al., 2021; Al-Sadi et al., 2015). Genetic determinants involved in conidial development, including hydrophobin-related genes such as *hfb4*, contribute to spore formation, dispersal, and resistance to environmental stress, thereby influencing storage performance (Cai et al., 2020). Likewise, adaptive responses involving heat shock proteins and other stress-responsive genes may enhance spore survival during prolonged storage (Ahmed et al., 2025; Mei et al., 2025).

Substrate composition also exerted a pronounced influence on fungal performance. Rice powder supported the highest initial spore production, confirming that finely milled cereal substrates rich in readily available nutrients promote rapid fungal colonization and sporulation. Their greater surface area and improved accessibility of carbohydrates may facilitate nutrient uptake and accelerate conidial production (Naeimi et al., 2020; Lima et al., 2024). Similarly, previous studies have shown that substrate composition, porosity, moisture retention, and aeration strongly influence fungal metabolism and propagule production during solid-state fermentation (Mulatu et al., 2021; Dhruw et al., 2023; Silva et al., 2024; Sala et al., 2021).

An interesting observation was the increase in spore viability between Day 10 and Day 30 before the gradual decline observed thereafter. This pattern suggests that fungal growth and conidiation continued during the early storage phase, probably because residual nutrients within the substrates remained available to support ongoing metabolic activity. Similar delayed sporulation has been reported in solid-state fermentation systems where fungal colonization continues after the initial incubation period, leading to increased propagule production during the first weeks of storage (Silva et al., 2024; Bilé et al., 2025). Likewise, *Trichoderma* species commonly reach maximum sporulation several weeks after inoculation before viability gradually declines as nutrients become depleted (Vitale et al., 2025; Locatelli et al., 2018). During the first month of storage, room temperature fluctuated between approximately 25 and 32 °C, while relative humidity ranged from 43% to 63%. These environmental conditions may also have favored continued metabolic activity and conidial maturation, as moisture availability and temperature strongly influence fungal physiological processes during storage (Bilé et al., 2025; Silva et al., 2024).

Following Day 30, spore viability declined progressively across all treatments, with significant reductions observed by Day 90. This decrease is consistent with the natural aging of fungal propagules and may be attributed to nutrient depletion, moisture changes, oxidative stress, and gradual deterioration of cellular structures (Naeimi et al., 2020; Fernández-Sandoval et al., 2012). Nevertheless, the stabilization of room temperature (26.5–27.0 °C) and relative humidity (72–74%) after the first month may have contributed to moderating further losses in viability by providing relatively stable storage conditions.

Although rice powder promoted superior initial sporulation, sorghum grain proved to be the most effective substrate for preserving spore viability during prolonged storage. Sorghum grain consistently maintained higher viable spore populations across isolates, particularly after 90 days. This superior performance is likely related to its favorable physical characteristics, including improved aeration, structural integrity, and a balanced nutrient supply. Whole-grain substrates provide a more stable microenvironment that protects propagules against desiccation and facilitates oxygen diffusion, whereas powdered substrates may compact during storage, limiting aeration and accelerating viability loss. Similar observations have been reported by Naeimi et al. (2020) and Kamboj et al. (2023), who found that sorghum grain supported greater formulation stability during extended storage. Conversely, although rice powder and sorghum powder enhanced early sporulation, their denser structure may have contributed to faster viability loss over time (Trinidad et al., 2025; Mulatu et al., 2021).

The significant interaction effects observed in this study further demonstrate that isolate performance cannot be explained by substrate or storage duration alone. The isolate × substrate interaction showed that a substrate's suitability depended on the isolate's physiological characteristics, while the isolate × storage duration and substrate × storage duration interactions indicated that the capacity to maintain viable propagules varied over time. Most importantly, the significant three-way interaction confirmed that storage stability resulted from the combined influence of isolate, substrate, and storage duration. Among the evaluated combinations, MG7-2 grown on sorghum grain maintained the highest spore viability after prolonged storage, whereas BH10-2 grown on rice powder exhibited one of the greatest reductions after 90 days. These findings suggest that isolate-specific traits, including stress tolerance, conidial wall composition, desiccation resistance, and metabolic adaptation, determine compatibility with specific substrates and ultimately influence the shelf life of formulations.

A limitation of this study is that substrate moisture content and water activity were not monitored throughout storage because the required analytical equipment was unavailable. Since both parameters strongly influence fungal metabolism and spore survival, future studies should quantify these variables to better explain changes in *Trichoderma* viability during storage.

Overall, the results demonstrate that maximizing the effectiveness of *Trichoderma*-based bioformulations requires consideration of both production efficiency and long-term storage stability. While rice powder is suitable for maximizing initial conidial production, sorghum grain provides superior preservation of spore viability during storage. Among the isolates evaluated, MG7-2 exhibited the greatest persistence during storage, indicating that the combination of MG7-2 and sorghum grain is the most promising formulation for developing stable and effective *Trichoderma*-based biocontrol products.

## 5 Conclusion and recommendation

The findings show that successful mass production of *Trichoderma* depends on optimizing both initial sporulation and long-term storage stability. Although rice powder produced the highest initial spore yield, sorghum grain provided the most favorable conditions for preserving spore viability during storage. The superior performance of isolate MG7-2, particularly when combined with sorghum grain, highlights the importance of matching fungal isolates to appropriate substrates to obtain bioformulations with extended life. These findings provide valuable guidance for selecting suitable substrates and isolates for the commercial production of *Trichoderma*-based biocontrol agents. Further studies should validate the efficacy of this formulation under field conditions, evaluate its storage stability beyond 90 days across different storage environments, and investigate alternative carrier materials and packaging technologies to improve the shelf life and commercial applicability of *Trichoderma* products.

## Data Availability

The original contributions presented in the study are included in the article/supplementary material, further inquiries can be directed to the corresponding author.
